# ROP-ET: a prospective phase III trial investigating the efficacy and safety of ropeginterferon alfa-2b in essential thrombocythemia patients with limited treatment options

**DOI:** 10.1007/s00277-024-05665-4

**Published:** 2024-03-04

**Authors:** Jean-Jacques Kiladjian, Francisca Ferrer Marin, Haifa Kathrin Al-Ali, Alberto Alvarez-Larrán, Eloise Beggiato, Maria Bieniaszewska, Massimo Breccia, Veronika Buxhofer-Ausch, Olga Cerna, Ana-Manuela Crisan, Catalin Doru Danaila, Valerio De Stefano, Konstanze Döhner, Victoria Empson, Joanna Gora-Tybor, Martin Griesshammer, Sebastian Grosicki, Paola Guglielmelli, Valentin García-Gutierrez, Florian H. Heidel, Arpád Illés, Ciprian Tomuleasa, Chloe James, Steffen Koschmieder, Maria-Theresa Krauth, Kurt Krejcy, Mihaela-Cornelia Lazaroiu, Jiri Mayer, Zsolt György Nagy, Franck-Emmanuel Nicolini, Francesca Palandri, Vassiliki Pappa, Andreas Johannes Reiter, Tomasz Sacha, Stefanie Schlager, Stefan Schmidt, Evangelos Terpos, Martin Unger, Albert Wölfler, Blanca Xicoy Cirici, Christoph Klade

**Affiliations:** 1 CIC 1427, Inserm, Université Paris Cité, 75010 Paris, France; 2 Centre d’Investigations Cliniques, AP-HP, Hôpital Saint-Louis, Paris, 75010 France; 3 Morales Meseguer University General Hospital, Regional Center of Blood Donation. CIBERER. UCAM. IMIB-Murcia, Murcia, Spain; 4 University Hospital Halle (Saale), Krukenberg Cancer Center Halle, Halle, Germany; 5 Department of Hematology, Hospital Clínic of Barcelona, Barcelona, Spain; 6 University Hospital City of Health and Science of Turin - Hospital Molinette, Complex Structure of Hematology, Torino, Italy; 7 Medical University of Gdańsk, Gdańsk, Poland; 8 Department of Translational and Precision Medicine, Sapienza University of Rome, Rome, Italy; 9 Department of Internal Medicine I for Hematology with Stem Cell Transplantation, Hemostaseology and Medical Oncology, Ordensklinikum Linz Elisabethinen, Johannes Kepler University Linz, Linz, Austria; 10 Medical Faculty, Johannes Kepler University Linz, Linz, Austria; 11 Clinic of Internal Hematology, University Hospital Kralovske Vinohrady, Prague, Czech Republic; 12 Fundeni Clinical Institute, Center for Hematology and Bone Marrow Transplantation, București, Romania; 13 Department of Clinical Hematology, Regional Institute of Oncology, Iasi, Romania; 14 Fondazione Policlinico Gemelli IRCCS, Section of Hematology, Catholic University, Rome, Italy; 15 Department of Internal Medicine III, University Hospital Ulm, Ulm, Germany; 16 AOP Health, Vienna, Austria; 17 Department of Hematooncology, Copernicus Memorial Hospital, Lodz, Poland; 18 Department of Hematology, Medical University of Lodz, Lodz, Poland; 19 Department of Oncology and Hematology, Ruhr University Bochum, Johannes Wesling Hospital Minden, Minden, Germany; 20 Medical University of Silesia, Katowice, Poland; 21 Department of Hematology, Careggi University Hospital, Florence, Italy; 22 Hospital Universitario Ramón y Cajal, Madrid (IRYCIS), Madrid, Spain; 23 Universidad de Alcalá, Madrid, Spain; 24 Clinic for Hematology, Hemostasis, Oncology and Stem Cell Transplantation, Hannover Medical School (MHH), Hannover, Germany; 25 Faculty of Medicine, Department of Internal Medicine, Division of Hematology, University of Debrecen, Debrecen, Hungary; 26 Ion Chiricuta Institute of Oncology, Hematology Department and Medfuture Research Center for Advanced Medicine, Iuliu Hatieganu University of Medicine and Pharmacy, Cluj-Napoca, Romania; 27 University Bordeaux, INSERM, BMC, U1034, F-33600 Pessac, France; 28 Laboratory of Hematology, Bordeaux University Hospital, Bordeaux, France; 29 Faculty of Medicine, Department of Hematology, Oncology, Hemostaseology, and Stem Cell Transplantation (Medical Clinic IV), RWTH Aachen University, Aachen, Germany; 30 Department of Internal Medicine I, Clinical Department of Hematology and Hemostaseology, Medical University of Vienna, Vienna, Austria; 31 Department of Hematology, Policlinica de Diagnostic Rapid Brasov, Brasov, Romania; 32 University Hospital Brno, Department of Internal Medicine, Hematology and Oncology, Masaryk University, Brno, Czech Republic; 33 Department of Internal Medicine and Hematology, Division of Hematology, Semmelweis University, Budapest, Hungary; 34 Centre Léon Bérard, Lyon, France; 35 IRCCS Azienda Ospedaliero-Universitaria di Bologna and Istituto di Ematologia Seràgnoli, Bologna, Italy; 36 University General Hospital Attikon, Athens, Greece; 37 Medical Clinic III, Hematology and Internistic Oncology, University Hospital Mannheim, Mannheim, Germany; 38 Department of Hematology, Jagiellonian University Hospital, Kraków, Poland; 39 Department of Internal Medicine V (Hematology and Oncology), Medical University Innsbruck, Innsbruck, Austria; 40 Department of Clinical Therapeutics, School of Medicine, National and Kapodistrian University of Athens, Athens, Greece; 41 Department of Internal Medicine, Clinical Divison of Hematology, Medical University Graz, Graz, Austria; 42 Institut Català d’ Oncologia- Hospital Germans Trias i Pujol, Josep Carreras Leukemia Research Institute, Universitat Autònoma de Barcelona, Barcelona, Spain

**Keywords:** Myeloproliferative neoplasms (MPNs), Essential thrombocythemia (ET), Ropeginterferon alfa-2b, ROP-ET, Phase III, Disease modification

## Abstract

**Abstract:**

Interferon-based therapies, such as ropeginterferon alfa-2b have emerged as promising disease-modifying agents for myeloproliferative neoplasms (MPNs), including essential thrombocythemia (ET). Current ET treatments aim to normalize hematological parameters and reduce the thrombotic risk, but they do not modify the natural history of the disease and hence, have no impact on disease progression. Ropeginterferon alfa-2b (trade name BESREMi®), a novel, monopegylated interferon alfa-2b with an extended administration interval, has demonstrated a robust and sustained efficacy in polycythemia vera (PV) patients. Given the similarities in disease pathophysiology and treatment goals, ropeginterferon alfa-2b holds promise as a treatment option for ET. The ROP-ET trial is a prospective, multicenter, single-arm phase III study that includes patients with ET who are intolerant or resistant to, and/or are ineligible for current therapies, such as hydroxyurea (HU), anagrelide (ANA), busulfan (BUS) and pipobroman, leaving these patients with limited treatment options. The primary endpoint is a composite response of hematologic parameters and disease-related symptoms, according to modified European LeukemiaNet (ELN) criteria. Secondary endpoints include improvements in symptoms and quality of life, molecular response and the safety profile of ropeginterferon alfa-2b. Over a 3-year period the trial assesses longer term outcomes, particularly the effects on allele burden and clinical outcomes, such as disease-related symptoms, vascular events and disease progression. No prospective clinical trial data exist for ropeginterferon alfa-2b in the planned ET study population and this study will provide new findings that may contribute to advancing the treatment landscape for ET patients with limited alternatives.

**Trial registration:**

EU Clinical Trials Register; EudraCT, 2023-505160-12-00; Registered on October 30, 2023.

**Supplementary Information:**

The online version contains supplementary material available at 10.1007/s00277-024-05665-4.

## Introduction

Recognized for their potential for disease modification, interferon-based therapies have gained recognition in myeloproliferative neoplasms (MPNs), a group of rare blood cancers [1]. Pegylated interferon alfa has shown good tolerability and efficacy in achieving hematologic remission in polycythemia vera (PV) patients [2–7] and to reduce or eliminate the need for phlebotomy [5, 7]. In addition to its ability to induce hematologic responses, pegylated interferon alfa triggers molecular remission by suppressing *JAK2*-driver mutation-carrying cells [2, 5, 7]. Robust and durable results were observed after long-term treatment [8, 9], coinciding with significantly less fluctuations in response and improved event-free survival compared with hydroxyurea (HU) or best available treatment, with risk events defined as thromboembolic events, disease progression, or mortality. Furthermore, some studies showed that sustained treatment with interferon alfa is associated with normalization of the bone marrow histopathology [10] and decreased risk of myelofibrosis, as compared to HU or phlebotomy alone [11]. This reflects the disease modifying activity of interferons through selective targeting of *JAK2*-mutated hematopoietic stem cells to induce exit from quiescence and promote terminal myeloid differentiation, resulting in the depletion of *JAK2*-mutant cells [12, 13].

Ropeginterferon alfa-2b, a unique formulation of pegylated interferon, is characterized by its favorable safety profile, long-term tolerability and convenient dosing schedules. Ropeginterferon alfa-2b was approved under the tradename BESREMi® in 2019 in Europe and in 2021 in US for the treatment of PV. Given the overlapping disease pathophysiology, clinical symptoms and treatment goals, ropeginterferon alfa-2b has emerged as a promising treatment option for other MPNs, especially essential thrombocythemia (ET) [14–16].

Approved treatments for ET, such as hydroxyurea (HU) and anagrelide (ANA), efficiently normalize hematological parameters, specifically platelet (HU and ANA) and white blood cell counts (HU), aiming to reduce the risk of vascular events [17, 18]. Due to teratogenic, carcinogenic, and/or potential leukemogenic risks HU and ANA may have limited suitability for specific patient subgroups. HU carries a high risk of non-melanoma skin cancer, a concern amplified by its often decades-long treatment course and patients with cardiovascular diseases or risk factors should be cautious with ANA due to potential cardiac side effects [19]. Alkylating agents such as busulfan (BUS) and pipobroman are effective cytoreductive options with availability in only a limited number of European countries and, owing to their leukemogenic potential, these agents are primarily considered secondary choices for elderly ET patients who are unresponsive or intolerant to other cytoreductive agents [20]. Importantly, none of these treatments possesses disease-modifying capabilities, thereby not addressing the risk of disease progression to secondary myelofibrosis or acute myeloid leukemia. Furthermore, resistance and/or intolerance to current treatments can occur in approximately 20–25% of patients with ET [21, 22]. Additionally, specific patient groups, such as younger patients are ineligible for available therapies due to contraindications because of potential teratogenic, carcinogenic, and leukemogenic risks (including HU, ANA, BUS). As a result, a significant portion of patients with ET are unable to receive available therapies, whether due to treatment failures, contraindications, or other safety concerns and are left with no approved treatment alternatives.

Therefore, the aim of this multicenter, prospective, single arm phase III trial is to provide evidence for ropeginterferon alfa-2b in the effective and safe management of ET patients lacking treatment options due to intolerance, resistance, and/or contraindications. The primary objective of this trial is to evaluate the capability of ropeginterferon alfa-2b to control abnormal hematologic parameters, disease-related symptoms and vascular risk, while controlling malignant mutation-carrying clones, thereby potentially slowing the neoplastic progression of the disease.

## Methods

### Study design and objectives

The ROP-ET trial is a multicenter, prospective, single-arm phase III trial designed to assess the use of ropeginterferon alfa-2b in the treatment of ET patients, who are intolerant, resistant, and/or not eligible for other cytoreductive treatments. The estimated total study duration per patient is a maximum of 36 months. This includes a 12-month period for primary analysis and a further 24 months to gather long-term data on efficacy and safety. Participating centers are listed in Table S1 (see Supplementary Information). The primary objective of the study is to assess the disease response rates of ropeginterferon alfa-2b in ET patients, defined by the modified ELN criteria as durable (for at least 3 months) peripheral blood count remission (platelets (PLTs) ≤ 400 × 10^9^/L and white blood cells (WBC) < 10 × 10^9^/L), absence of hemorrhagic or thrombotic events and disease progression, durable improvement and non-progression in disease-related signs, and durable symptoms improvement based on the Myeloproliferative Neoplasm Symptom Assessment Form Total Symptom Score (MPN-SAF TSS). The secondary objectives include to further assess the efficacy of ropeginterferon alfa-2b in terms of disease response, symptom improvement, vascular events, disease progression, and quality of life (QoL). Additionally, the study aims to evaluate the efficacy of ropeginterferon alfa-2b with regard to disease modification, defined by a sustained decline in mutant allele burden of the driver mutations *JAK2, CALR*, or *MPL*. Safety and tolerability of ropeginterferon alfa-2b is planned to be assessed in the study population throughout the entire study duration.

### Study population

The inclusion and exclusion criteria for this study are outlined in Table 1. Major eligibility criteria include male or female patients aged 18 or older diagnosed with ET according to WHO 2016 criteria [23]. The diagnosis is to be confirmed by a bone marrow biopsy not more than 5 years old. The study focusses on patients who require cytoreductive treatment but are intolerant, resistant to, and/or ineligible for all locally approved cytoreductive therapies for the treatment of ET, such as HU, ANA, BUS, and pipobroman. Resistance or intolerance to HU should be documented according to the modified ELN criteria [24]. Resistance or intolerance to ANA, BUS, or pipobroman is defined based on the non-responder status according to the primary efficacy endpoint of the study protocol or by the presence of treatment-related toxicities. Patients are considered ineligible for HU, ANA, BUS, or pipobroman due to contraindications according to locally available product information or due to investigator-assessed benefit-risk concerns. Additionally, patients must be interferon-treatment naive, and if they have received prior cytoreductive treatment, a washout period of at least 14 days or longer is required.

**Table 1:** Eligibility criteria for ROP-ET study

Inclusion criteria	Exclusion criteria
1. Informed consent 2. Age ≥ 18 years 3. ET diagnosis (WHO 2016 criteria) with a bone marrow biopsy test result not more than 5 years old 4. Need for cytoreductive treatment and documented resistance/intolerance to, and/or ineligibility for all cytoreductive therapies locally registered for the treatment of ET in the EU (i.e., HU, ANA, BUS, and pipobroman) **§** 5. If patient received any prior cytoreductive treatment for ET, the washout period between the last dose of treatment and the first dose of study drug must be at least 14 days, or longer. (If washout period was not completed at first day of patients screening, washout may be done after obtaining ICF during the 28-day screening phase) 6. Interferon treatment-naive 7. Adequate hepatic function defined as bilirubin ≤ 1.5 x ULN, prothrombin time (INR) ≤ 1.5 x ULN, albumin > 3.5 g/dL, alanine aminotransferase ≤ 2.0 x ULN, aspartate aminotransferase ≤ 2.0 x ULN at screening 8. HADS score 0–7 on both subscales 9. Patient with HADS score of 8–10 inclusive on either, or both, of the subscales may be eligible following psychiatric assessment that excludes clinical significance of the observed symptoms in the context of potential treatment with an interferon alfa.	1. Any patient requiring a legally authorized representative 2. Any hypersensitivity to interferon alfa or to any of the drug excipients 3. Pre-existing thyroid disease, if not in remission or not controlled with conventional treatment 4. Existence of, or history of severe psychiatric disorders, particularly severe depression, suicidal ideation or suicide attempt 5. Severe cardiovascular disease (i.e. uncontrolled hypertension, congestive heart failure (≥ NYHA class 2), serious cardiac arrhythmia, significant coronary artery stenosis, unstable angina) or recent stroke or myocardial infarction or pulmonary hypertension 6. Patients with diabetes mellitus that cannot be effectively controlled by medicinal products 7. History or presence of autoimmune disease (excluding well-controlled Hashimoto’s disease) 8. Immunosuppressed transplant recipients 9. Concomitant treatment with telbivudine 10. Decompensated cirrhosis of the liver (Child-Pugh B or C) 11. End stage renal disease (GFR < 15 mL/min) 12. Symptomatic splenomegaly (per the investigator’s judgement) 13. Patients with any other significant medical conditions that, in the opinion of the investigator, would compromise the results of the study or may impair compliance with the requirements of the protocol¶ 14. Use of any investigational drug < 4 weeks prior to the first dose of study drug, or ongoing effects/symptoms due to prior administration of any investigational agent 15. HADS score of 11 or higher on either, or both, of the subscales, and /or development or worsening of the clinically significant depression or suicidal thoughts 16. Pregnant patients, breastfeeding patients or females of childbearing potential not willing to comply with contraceptive requirements
**§ Patients resistant/intolerant to HU** must have documented resistance/intolerance as defined by modified ELN criteria [24], whereby at least one of the following criteria is met: • Platelet count > 600 × 10^9^/L at ≥ 2 g/day (or ≥ 2.5 g/day if patient body weight > 80 kg) or maximally tolerated dose if < 2 g/day or at maximum dose per local practice after at least 3 months of HU • Platelet count > 400 × 10^9^/L and WBC count < 2.5 × 10^9^/L at any dose and any duration of HU • Platelet count > 400 × 10^9^/L and Hb < 10 g/dL at any dose and any duration of HU • Presence of HU-related toxicities at any dose and any duration of therapy (e.g. leg ulcers, mucocutaneous manifestations, pneumonitis, or HU-related fever) **Patients resistant/intolerant to ANA, BUS or pipobroman** must meet one of the following criteria: • Patient designated as non-responder according to the primary efficacy endpoint of this protocol (modified ELN criteria) after at least 3 months of treatment with the recommended dosing defined in SmPC or local practice • Presence of treatment-related toxicities at any dose and any duration of therapy **Patients ineligible for HU, ANA, BUS or pipobroman** with contraindications as defined by locally available SmPC or designated as such by investigator due to benefit-risk concerns	¶ including but not limited to: • History of any malignancy within 5 years (except stage 0 chronic lymphocytic leukemia, basal cell, squamous cell, and superficial melanoma) • Infections with systemic manifestations (e.g. bacterial, fungal, or HIV, except HBV and/or HCV, at screening) • Evidence of severe retinopathy (e.g. cytomegalovirus retinitis, macular degeneration) or clinically relevant ophthalmological disorder (due to diabetes mellitus or hypertension) • History of alcohol or drug abuse within the last year

*ANA *Anagrelide; BUS, Busulfan, *ET *Essential Thrombocythemia, *GFR *Glomerular Filtration Rate, *HADS *Hospital Anxiety and Depression Scale, *HBV *Hepatitis B virus, *HCV *Hepatitis C virus, *Hb *Hemoglobin, *HIV *Human immunodeficiency virus, *HU *Hydroxyurea, *ICF *Informed consent form, *INR *International Normalized Ratio, *NYHA *New York Heart Association, *SmPC* Summary of Product Characteristics, *ULN* Upper Limit of Normal, *WBC *White Blood Cell

### Study drug

Ropeginterferon alfa-2b will be administered as a subcutaneous injection every two weeks for up to 36 months at a dose of 125 µg per injection. The dose may be adjusted to 250 or 500 µg every two weeks only if optimal hematologic response is not achieved after 3 months or after 6 months, respectively. Treatment may be interrupted, or the dose reduced in case of drug-related toxicity or intolerance. After 12 months, dosing frequency may be adjusted to every four weeks at the discretion of the investigator. Low-dose aspirin (75–100 mg/day) may be given, unless contraindicated.

### Study assessments

**Fig. 1:**
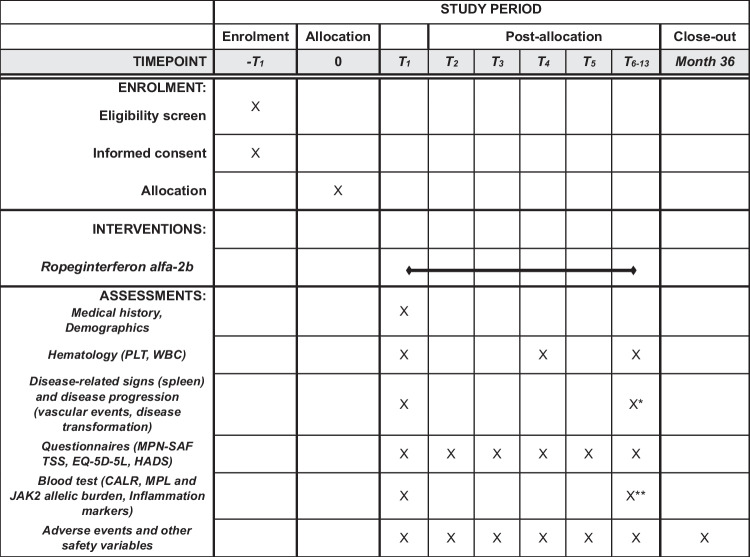
Schedule of enrolment and assessments (SPIRIT 2013 Figure). T1: Baseline assessments, start of treatment, T2: 2 wks after treatment start, T3: 4 wks after treatment start, T4: 6 wks after treatment start, T5: 8 wks after treatment start, T6-13: 3, 6, 9,12, 18, 24, 30, 36 months after treatment start; *except T6; **except T6 and T8. *Abbreviations:* CALR, Calreticulin; EQ-5D-5L, EuroQol 5-Dimensions 5-Levels; HADS, Hospital Anxiety and Depression Scale; JAK2, Janus Kinase 2; MPL, Myeloproliferative Leukemia Virus Oncogene; MPN-SAF TSS, Myeloproliferative Neoplasm Symptom Assessment Form Total Symptom Score; PLT, Platelet; WBC, White Blood Cell

 The estimated total study duration per patient is maximum 36 months, which includes 12 months for primary analysis. Detailed study procedures can be found in the SPIRIT figure (Fig. 1). Patient visits will be scheduled every two weeks in the first two months, followed by visits every 3 months until month 12, and visits every 6 months thereafter. Disease response assessments will be performed every 3 months in the first year of treatment, and every 6 months in the second and third year of treatment. Primary efficacy is evaluated at 12 months and includes assessments of peripheral blood counts (PLT and WBC; performed by a central laboratory), ET-related hemorrhagic or thrombotic events, disease progression (i.e. transformation into PV, post-ET myelofibrosis, myelodysplastic syndrome or acute leukemia), disease-related signs (splenomegaly) and MPN-SAF TSS. Quantitative measurements of *JAK2*, *CALR*, and *MPL* allelic burden will be undertaken by a central laboratory every 6 months. The assessment of safety will include monitoring of vital signs, clinical safety laboratory tests, physical examinations, ECG evaluation, ECOG performance status, ocular examination, and adverse events.

### Outcomes

The primary efficacy endpoint of the study will assess the rate of disease response at month 12, utilizing modified criteria established by a consensus of the working group comprising the European LeukemiaNet (ELN) and International Working Group-Myeloproliferative Neoplasms Research and Treatment (IWG-MRT) [25]. Disease response for the primary efficacy endpoint will be defined as follows: (i) durable peripheral blood count remission (PLTs ≤ 400 × 10^9^/L and WBCs < 10 × 10^9^/L) lasting for at least 3 months, (ii) absence of hemorrhagic or thrombotic events and disease progression, (iii) durable improvement or non-progression in disease-related signs, and (iv) durable symptom improvement or maintenance of non-progression based on the MPN-SAF TSS. Progression of disease-related signs is defined as the conversion from asymptomatic to symptomatic splenomegaly or a clinically relevant progression of spleen size at the discretion of the investigator. The definition of symptom improvement or maintenance of non-progression is based on changes from baseline MPN-SAF TSS with changes defined as follows: for baseline scores ≥ 20, a 10-point score reduction; for baseline scores inclusive of 15–19, a 5-point score reduction; for baseline scores inclusive of 10–14, a score decrease to ≤ 10 points; and for a baseline score < 10, the score stays < 10. Secondary endpoints include response rates according to the modified ELN criteria at months 9, 18, 24, 30, and 36, longitudinal changes in response rates over 12 months, additional response analyses (e.g. time and duration of first disease response or first peripheral blood count remission response), occurrence of thromboembolic and bleeding events and disease progression, assessment of QoL and symptomatic improvement (assessed using the EQ-5D-5 L questionnaire and the MPN-SAF TSS, respectively), change of inflammation markers (CRP, pentraxin 3, GRO-α, EGF) and cytokines (BLC, M-CSF, eotaxin-2, and TIMP-1) and change of *JAK2*, *CALR*, or *MPL* allelic burden over time. As an exploratory aspect of the study, patients have the option to participate in a sub-study focused on neutrophil extracellular traps (NETs) marker testing. The purpose of this sub-study is to investigate the impact of ropeginterferon alfa-2b on plasma NETs and its association with thrombotic events, and the measurement will include the assessment of circulating cell-free DNA (as a non-specific marker of NETs) and citH3-DNA complexes (as a specific marker of NETs).

Adverse events are graded according to Common Terminology Criteria for Adverse Events (CTCAE; version 5.0) and captured throughout the study.

### Sample size

 Based on previous studies using ELN response criteria for disease response assessment [4, 26] and the proposed inclusion and exclusion criteria, a response rate of at least 40% for ropeginterferon alfa-2b in the overall population is anticipated. To achieve a level of precision of 10%, the estimated sample size for the primary endpoint analysis is 93 patients. Assuming a 20% dropout rate, a total of 117 patients will be enrolled in the study to ensure that 93 patients remain evaluable for primary endpoint assessment at 12 months. The two subgroups of interest are patients below the age of 45 and cytoreductive treatment resistant/intolerant patients. Assuming 40% response rate and approximately 47 patients evaluable for primary endpoint assessment at 12 months in each subgroup, level of precision will be 13.5%.

### Statistical analysis methods

 The statistical analysis of this clinical trial will adhere to the guidelines outlined in ICH E9, Statistical Principles for Clinical Trials [27]. A finalized statistical analysis plan will be developed prior to study start (i.e. inclusion of the first patient). For continuous data, the descriptive statistics include the number of cases, mean, standard deviation, median, lower quartile (Q1), upper quartile (Q3), minimum, and maximum. Statistical analyses will be performed using Statistical Analysis System (SAS®) software, version 9.4 or higher (SAS Institute, NC, USA).

The primary efficacy endpoint is the durable disease response rate at month 12. An interim analysis of the 12-month primary endpoint is planned to be performed after the last patient had reached month 12. The number and percentage of patients with a durable disease response at month 12 will be calculated, and the 95% confidence interval for the rate will be estimated using the Clopper-Pearson method. The median and 95% confidence interval of the time-to-event end points will be estimated using the Kaplan–Meier method. For response assessment, patients prematurely discontinuing due to safety or efficacy reasons before the time-point of interest will be considered non-responders. Subgroups of interest are predefined and include patients below the age of 45 and cytoreductive treatment resistant/intolerant. Additional subgroup and sensitivity analyses will have exploratory character and will be defined in all details in the statistical analysis plan. All safety data will be analyzed descriptively. Adverse events will be categorized by system organ classes and preferred terms as defined by the MedDRA dictionary and will be graded using CTCAE v.5.0. Descriptive statistics will be calculated for the number of occurrences, the number of patients, and the incidence of various types of adverse events, summarised by its seriousness, severity, and relationship to the drug.

## Discussion

 Interferons are well established in the management of patients with PV and are commonly used off-label in the treatment of other MPNs, especially ET [28–34]. Current guidelines recommend interferon alfa as first- and second-line treatment in high-risk ET patients as well as in younger patients, including those who are pregnant or planning pregnancy [35–37]. Meta-analyses of large cohorts of patients with ET reveal that interferon alfa exhibit high clinical efficacy with an overall response rate (ORR) exceeding 80% and with approximately 60% achieving complete hematologic remission (CHR) [38, 39]. In addition, treatment of ET with interferon alfa induces a molecular response in 42% of patients (95% CI: 31–52%) [39] and a significant improvement in myelofibrosis-free survival [40], suggesting that, much like in PV, interferons hold the potential for disease modification in patients with ET.

In patients with ET switching from other cytoreductive therapies, clinical evidence confirms the efficacy and safety of pegylated interferon as second- or third-line therapy option, as summarized in Table 2. Toxicity-related discontinuation rates ranged from 11 to 28% in studies reporting discontinuation rate due to adverse events among ET patients [4, 26, 41–43]. Overall, an ORR in the ranges of 69–100% and 54–78% were observed based on hematologic response and composite hematologic and symptoms response, respectively. Of note, the variability in response rates to interferon for ET arises from using various formulations (peg-IFN-α-2a/b, ropeg-IFN-α-2b), broad inclusion criteria (new and pretreated patients) and varied response criteria definitions. While providing promising clinical evidence for ropeginterferon alfa-2b in treating patients with ET, including those who have undergone and/or failed prior cytoreductive therapies, available studies, however, have several limitations. Most are from single or a few academic centers, involve small participant numbers, have short follow-up periods (up to 2 years), or are retrospective in design. Assessments of clinical outcomes such as vascular events, disease progression, symptom relief, and improvements in quality of life were not frequently conducted in these studies. Yacoub et al. reported that in response to interferon therapy patients experienced significant improvements in MPN-related symptoms, notably in fatigue, dizziness, numbness and tingling, and weight loss [4]. Furthermore, the impact of interferon on mutational burden was assessed in only a few studies [2, 4, 26, 43] with observed molecular response rates of 37% for *JAK2*V617F and 42% for *CALR* allele burden [2, 43] and longitudinal evaluations showing a reduction in *JAK2*V617F variant allele frequency from 30.5 to 8.3% over 4 years and 18.1% after 8 years [26]. Of note, these analyses are mostly based on results from a subset of patients only, depending on sample availability and baseline mutational status.

**Table 2:** Clinical studies with pegylated interferon alfa in ET patients with prior cytoreductive therapies

Reference	Study design; follow-up	Treatment*	n of ET patients	Patient population	Previous treatment	Outcomes	Molecular response	Outcome definition	Discontinuation due to toxicity, n (%)
[41]	Prospective Single center FU: NA	Peg-IFN alpha 2b 3 µg/kg/wk	11	New diagnosis and previously treated	Cytoreduction naive: 4/11 Cytoreduction treated: 7/11	ORR: 100% (CHR: 100%, PHR: 0%)	NA	CHR: platelet count < 400 × 10^9^/L, no TE events PHR: platelet count 400–600 × 10^9^/L, no TE events	2 (18.2%)
[44]	Retrospective Multicenter FU: 1.4 years	Peg-IFN alpha 2a 80 µg/wk	46	New diagnosis and previously treated	NA	ORR: 78% (CR: 63%; PR: 15%)	NA	2009 ELN response criteria	Not reported separately for ET patients
[45]	Retrospective Single center FU: 3.8 years	Peg-IFN alpha 2a 45 µg/wk	20	New diagnosis and previously treated	NA	ORR: 65% (CR: 25%, PR: 20%, CR/PR: 20%)	NA	2013 ELN/IWG-MRT response criteria	Not reported separately for ET patients
[46]	Prospective Single center FU: 2.3 years	Peg-IFN alpha 2b 2 µg/kg/wk	13	New diagnosis and previously treated	NA	ORR: 69% (CHR: 54%; PHR: 15%)	NA	CHR: platelet count < 440 × 10^9^/L PHR: >50% reduction of platelet count without achieving normal levels	Not reported separately for ET patients
[42]	Prospective Multicenter FU: 2 years	Peg-IFN alpha 2b 50 µg/wk	36	High-Risk ET, New diagnosis and previously treated	Cytoreduction naive: 28/36 Cytoreduction treated: 8/36	ORR: 75% (CHR: 67%, PHR: 8%)	NA	CHR: platelet count < 450 × 10^9^/L PHR: platelet count 450–600 × 10^9^/L	10 (28%)
[2]	Prospective Single center FU: 7 years	Peg-IFN alpha 2a 90 µg/wk	40	New diagnosis and previously treated	NA	ORR: 80% (CHR: 73%; PHR: 3%)	Baseline: *JAK2*V617F *n* = 19 (48%); median allelic burden: 23% ORR: 37% (CMR: 9%, PMR: 17%)	CHR/PHR based on 2009 ELN response criteria CMR: defined as *JAK2*V617F ULD, PMR as reductions in baseline allele burden of > 50%	Not reported separately for ET patients
[47]	Prospective Multicenter FU: 2 years	Peg-IFN alpha 2b 0.5 µg/kg/wk	21	New diagnosis and previously treated	NA	CHR: 33%	NA	CHR: platelet count < 400 × 10^9^/L if previous TE events or platelet count < 600 × 10^9^/L if no previous TE events	Not reported separately for ET patients
[26]	Retrospective Multicenter FU: 2.3 years	Peg-IFN alpha 2a and b no information on dosing	127	New diagnosis and previously treated	Cytoreductive naive: 75/127 Cytoreductive treated: 52/127	ORR: 89% (CHR: 54%, PHR: 35%)	Baseline: *JAK2*V617F (51%); median allelic burden: 30.5% allelic burden reduced to 8.3% after 4 years, and 18.1% after 8 years (based on *n* = 14)	CHR/PHR based on 2009 ELN response criteria	16%
[43]	Prospective Observational Multicenter FU: 12 years	Peg-IFN alpha 124 µg/wk	31	New diagnosis and previously treated; *CALR*mut+	Cytoreductive naive: 6/31 Cytoreductive treated: 25/31	ORR: 100% (CHR: 77%, PHR: 23%)	Baseline: *CALR*mut (*n* = 31) median allelic burden: 41% (baseline) reduced to 26% ORR: 42%; (CMR: 6%, PMR 36%)	CHR/PHR based on 2009 ELN response criteria CMR: defined as CALR ULD, PMR as reductions in baseline allele burden of > 50%	19%
[4]	Prospective Multicenter FU: 1.6 years	Peg-IFN alpha 2a 45 µg/wk	65	High risk ET, HU resistant/intolerant	Cytoreductive treated:65/65	ORR: 69% (CR 43%,26% PR)	Baseline: *JAK2*V617F *n* = 31 (48%), median allele burden: 29% median absolute reduction in *JAK2*V617F VAF was 6% (range: -84–47%) in patients achieving a CR vs. 4% (range: -18–56%) in patients with PR or no response	2009 ELN response criteria	7 (11%)

* Interferon doses were modified based on patients’ individualized response and tolerance, only the starting or median/mean doses are displayed

*CHR *Complete hematologic response, *CMR *Complete molecular response, *CR *Complete remission, *ELN *European Leukemia Network, *ET *Essential thrombocytemia, *FU* Median or mean follow-up period (in years), *IFN *Interferon, *IWG-MRT *International Working Group for Myeloproliferative Neoplasms Research and Treatment, *NA *Not available, *ORR *Overall response rate, *PHR *Partial hematologic response, *PR *Partial remission, PMR Partial molecular response, *TE *Thromboembolic events, *ULD *Under the level of detection, *VAF *Variant allele frequency

Source: [38, 39]

Larger, prospective studies are warranted in cohorts of patients who require cytoreductive therapy but lack alternative treatment options, focusing particularly on the impact of interferon alfa on molecular response and disease modification, validating preliminary findings and elucidating potential clinical significance. Ropeginterferon alfa-2b is currently being studied in ongoing trials for the treatment of ET: The Surpass-ET trial (NCT04285086) focuses on high-risk ET patients that are resistant or intolerant to HU [48], while the Exceed-ET trial (NCT05482971) includes treatment-naive as well HU and ANA pretreated patients. In addition to enrolling cytoreductive treatment resistant and intolerant patients, the ROP-ET trial distinctively includes those who are ineligible or contraindicated for approved therapies, thereby addressing a critical gap in the currently available care and treatment of ET patients. Ongoing trials, similar to the ROP-ET trial, plan the primary endpoint analysis after 12 months. Additionally, for the Exceed-ET trial there is an extension of up to 3 years for responding patients [49], while the ROP-ET trial is designed with a 3-year treatment duration for all enrolled patients. As demonstrated in pivotal trials that contributed to the approval of ropeginterferon alfa-2b for PV [9, 50] as well as a recently published clinical trial [6], response to pegylated interferon alfa deepens with longer treatment duration and hence, a significant treatment duration is necessary to achieve disease response, particularly on a molecular level. The planned treatment duration in the ROP-ET trial allows to evaluate long-term outcomes and a comprehensive assessment of the treatment impact on clinical and molecular response. Maintaining patients on treatment for a sufficient time to achieve possible disease modification necessitates a dosing regimen that is well-tolerated. Notably, the highest treatment-related discontinuation rates for pegylated interferon alfa occur during the initial treatment phase [2, 9, 26]. Our dosing strategy, therefore, adopts a low starting dose (125 µg) that is anticipated to be an effective and well-tolerated maintenance dose for the majority of patients, as evidenced by platelet response [7] and long-term tolerability [8]. If the desired disease response (CHR) is not achieved, an individualized dose escalation to 250 or 500 µg can be employed, reinforcing that these higher doses are alternative strategies for poor responders and not a target for all. This low-dose run-in concept has led to the approval of ropeginterferon alfa-2b by EMA and FDA and is confirmed by recently published study results from other investigators, ensuring improved drug tolerability (with discontinuation rates at or below 10%), while achieving a timely and favorable therapeutic response [7, 50, 51].

Chronic inflammation is a hallmark characteristic of MPNs and plays a significant role in disease progression [52–54]. In addition, inflammatory cytokines in MPN patients are involved in the interplay between inflammation and thrombosis, the so-called MPN thromboinflammation. *JAK2*V617F, the most prevalent mutation in ET, has been shown to activate not only erythrocytes and platelets, but also granulocytes. Specifically, activation of neutrophils leads to an increased NETs generation [55, 56], which are extracellular net-like structures containing DNA, proteases and enzymes, such as myeloperoxidase and neutrophil elastase. Data regarding NETosis in MPNs and its association with thrombotic events are conflicting [57] and no comprehensive analysis is available investigating the impact of interferon alfa therapy on NETosis in a large cohort of ET patients [55, 56, 58, 59]. Hence, the ROP-ET trial is designed to assess the effects of ropeginterferon alfa-2b on cytokine profiles and NET markers in ET patients with the goal to explore and better understand the relationship between these markers and thrombotic events.

In summary, the ROP-ET trial is a multicenter, prospective, single-arm phase III trial in ET patients with highly limited treatment options for whom ropeginterferon alfa-2b represents a promising cytoreductive therapy with proven long-term safety and tolerability. The primary objective is to assess the safety of ropeginterferon alfa-2b in ET patients with resistance, intolerance and/or ineligibility to currently available therapies and to demonstrate its ability to regulate abnormal hematologic parameters and disease symptoms while depleting malignant driver mutation-carrying clones, thereby altering the natural progression of the disease.

### Supplementary information

Below is the link to the electronic supplementary material.

ESM 1 (DOCX 37.2 KB (38)

ESM 2 (PDF 189 KB)

## Data Availability

No datasets were generated or analysed during the current study.

## Declarations

### Trial Status

Protocol Version 3.0 dated 26 Sep 2023. The first subject was enrolled on 04 Dec 2023.
