# Supplementary material for: ROP-ET: a prospective phase III trial investigating the efficacy and safety of ropeginterferon alfa-2b in essential thrombocythemia patients with limited treatment options
Source: Ann Hematol. 2024 Mar 4;103(7):2299–310. doi: 10.1007/s00277-024-05665-4 (PMC11224110; doi:10.1007/s00277-024-05665-4)
Supplement: Supplementary file 1 — (DOCX 37.2 KB (38) [file 277_2024_5665_MOESM1_ESM.docx]

# Supplemental Appendix

**Article title: “**ROP-ET: A Prospective Phase III Trial Investigating the Efficacy and Safety of Ropeginterferon Alfa-2b in Essential Thrombocythemia Patients with Limited Treatment Options”

**Journal name:** Annals of Hematology

**Author names:** Jean-Jacques Kiladjian ^1^, Francisca Ferrer Marin ^2^, Haifa Kathrin Al-Ali ^3*^, Alberto Alvarez-Larrán ^4*^, Eloise Beggiato ^5*^, Maria Bieniaszewska ^6*^, Massimo Breccia ^7*^, Veronika Buxhofer-Ausch ^8*^, Olga Cerna ^9*^, Ana-Manuela Crisan ^10*^, Catalin Doru Danaila ^11*^, Valerio De Stefano ^12*^, Konstanze Döhner ^13*^, Victoria Empson ^14^, Joanna Gora-Tybor ^15*^, Martin Griesshammer ^16*^, Sebastian Grosicki ^17*^, Paola Guglielmelli ^18*^, Valentin García-Gutierrez ^19*^, Florian H. Heidel ^20*^, Arpád Illés ^21*^, Ciprian Tomuleasa ^22*^, Chloe James ^23*^, Steffen Koschmieder ^24*^, Maria-Theresa Krauth ^25*^, Kurt Krejcy ^14^, Mihaela-Cornelia Lazaroiu ^26*^, Jiri Mayer ^27*^, Zsolt György Nagy ^28*^, Franck-Emmanuel Nicolini ^29*^, Francesca Palandri ^30*^, Vassiliki Pappa ^31*^, Andreas Johannes Reiter ^32*^, Tomasz Sacha ^33*^, Stefanie Schlager ^14^, Stefan Schmidt ^34*^, Evangelos Terpos ^35*^, Martin Unger ^14^, Albert Wölfler ^36*^, Blanca Xicoy Cirici ^37*^ and Christoph Klade ^14^

**Corresponding author:** Prof. Jean-Jacques Kiladjian, MD PhD, Université Paris Cité, CIC 1427, Inserm, 75010 Paris, France and Centre d’Investigations Cliniques, AP-HP, Hôpital Saint-Louis, 75010 Paris, France.

Email: [jean-jacques.kiladjian@aphp.fr](mailto:jean-jacques.kiladjian@aphp.fr).

Table S1 List of participating centers

| **Country** | **Principal Investigator** | **Address** |
| --- | --- | --- |
| Austria | Veronika Buxhofer-Ausch | Ordensklinikum Linz Elisabethinen, Department of Internal Medicine I for Hematology with Stem Cell Transplantation, Hemostasis and Medical Oncology,  Fadingerstrasse 1  4020 Linz |
| Austria | Maria-Theresa Krauth | Medical University Vienna, Department of Internal Medicine I, Clinical Division of Hematology and Hemostaseology  Waehringer Guertel 18-20  1090 Vienna |
| Austria | Stefan Schmidt | Medical University Innsbruck, Department of Internal Medicine V (Haematology and Oncology)  Anichstrasse 35  6020 Innsbruck |
| Austria | Albert Wölfler | Medical University Graz, Department of Internal Medicine, Clinical Divison of Hematology  Auenbruggerplatz 38  8036 Graz |
| Czech Republic | Olga Cerna | University Hospital Kralovske Vinohrady, Clinic of Internal Haematology  Srobarova 1150/50  Praha 10  100 34 Prague |
| Czech Republic | Jiri Mayer | University Hospital Brno, Department of Internal Medicine, Hematology and Oncology  Jihlavska 20  625 00 Brno |
| France | Chloe James | Université Bordeaux, INSERM, BMC, U1034, F-33600 Pessac, France CHU Bordeaux, Laboratoire d’hématologie, Hopital Haut Leveque, 1 av de Magellan, Pessac, France |
| France | Jean-Jacques Kiladjian | Hôpital Saint-Louis, Centre d’Investigations Cliniques  1, Avenue Claude Vellefaux  75010 Paris |
| France | Franck-Emmanuel Nicolini | Centre Léon Bérard  28 rue Laennec  69373 Lyon |
| Germany | Haifa Kathrin Al-Ali | University Hospital Halle (Saale), Krukenberg Cancer Center Halle  Ernst-Grube-Strasse 40  06120 Halle |
| Germany | Konstanze Döhner | University Hospital Ulm, Department of Internal Medicine III  Albert-Einstein-Allee 23  89081 Ulm |
| Germany | Martin Griesshammer | Johannes Wesling Hospital Minden, Department of Oncology and Hematology  Hans-Nolte-Straße 1  32429 Minden |
| Germany | Florian Heidel | Hannover Medical School (MHH), Clinic for Hematology, Hemostasis, Oncology and Stem Cell Transplantation  Carl-Neuberg-Str. 1  30625 Hannover |
| Germany | Steffen Koschmieder | RWTH Aachen University, Faculty of Medicine, Department of Hematology, Oncology, Hemostaseology, and Stem Cell Transplantation (Medical Clinic IV)  Pauwelsstrasse 30  52074 Aachen |
| Germany | Andreas Johannes Reiter | University Hospital Mannheim, Medical Clinic III, Hematology and Internistic Oncology  Theodor-Kutzer-Ufer 1-3  68167 Mannheim |
| Greece | Vassiliki Pappa | University General Hospital Attikon  1 Rimini Str.,  Chaidari,  12462 Athens |
| Greece | Evangelos Terpos | Department of Clinical Therapeutics, National and Kapodistrian University of Athens, School of Medicine  78 Vassilissis Sophias Avenue  11528 Athens |
| Hungary | Arpád Illés | University of Debrecen, Faculty of Medicine, Department of Internal Medicine, Division of Hematology  Nagyerdei krt. 98.  4032 Debrecen |
| Hungary | Zsolt György Nagy | Semmelweis University, Department of Internal Medicine and Haematology, Division of Hematology  Szentkiralyi utca 46  1088 Budapest |
| Italy | Eloise Beggiato | University Hospital City of Health and Science of Turin - Hospital Molinette, Complex Structure of Hematology  Corso Bramante 88  10126 Turin |
| Italy | Massimo Breccia | Sapienza University of Rome, Department of Translational and Precision Medicine  Via Policlinico 155  00161 Rome |
| Italy | Valerio De Stefano | Fondazione Policlinico Gemelli IRCCS, Section of Hematology  Largo Agostino Gemelli 8  00168 Rome |
| Italy | Paola Guglielmelli | Careggi University Hospital, Department of Hematology  Largo Brambilla 3  50134 Florence |
| Italy | Francesca Palandri | IRCCS Azienda Ospedaliero-Universitaria di Bologna  Via Massarenti 9  40138 Bologna |
| Poland | Maria Bieniaszewska | Medical University of Gdańsk, Dept. of Haematology and Transplantology  Mariana Smoluchowskiego 17  80-214 Gdansk |
| Poland | Joanna Gora-Tybor | Nicolaus Copernicus Memorial Hospital, Department of Hematooncology  ul. Pabianicka 62  93-513 Lodz |
| Poland | Sebastian Grosicki | Medical University of Silesia  ul. Tadeusza Kościuszki 92  40-519 Katowice |
| Poland | Tomasz Sacha | Jagiellonian University Hospital, Department of Hematology  2 Jakubowskiego St.,  30-688, Krakow |
| Romania | Ana-Manuela Crisan | Fundeni Clinical Institute, Center for Hematology and Bone Marrow Transplantation  Șoseaua Fundeni 258  București 022328 |
| Romania | Catalin Doru Danaila | Iasi Regional Institute of Oncology, Department of Hematology  Str. General Berthelot2-4  700483 Iasi |
| Romania | Mihaela-Cornelia Lazaroiu | Policlinica de Diagnostic Rapid Brasov, Department of Hematology Strada Carierei 65A  500052 Brasov |
| Romania | Ciprian Tomuleasa | Ion Chiricuta Institute of Oncology / Iuliu Hatieganu University of Medicine and Pharmacy, Hematology Department  34-36 Republicii Street  400015 Cluj-Napoca |
| Spain | Alberto Alvarez-Larrán | Hospital Clínic of Barcelona, Department of Hematology  Calle Villarroel 170  08036 Barcelona |
| Spain | Francisca Ferrer Marin | Morales Meseguer University General Hospital, Regional Center of Blood Donation  Marques de los Velez Ave.  30008 Murcia |
| Spain | Valentin García-Gutierrez | Hospital Universitario Ramón y Cajal, Instituto Ramón y Cajal de Investigación Sanitaria (IRYCIS)  Ctra. de Colmenar Viejo km. 9, 100  28034 Madrid |
| Spain | Blanca Xicoy Cirici | University Hospital Germans Trias i Pujol, Department of Clinical Hematology  Carretera de Canyet s/n  08916 Badalona |
